# Combined use of stress echocardiography and cardiopulmonary exercise testing to assess exercise intolerance in patients treated for acute myocardial infarction

**DOI:** 10.1371/journal.pone.0255682

**Published:** 2021-08-05

**Authors:** Krzysztof Smarz, Tomasz Jaxa-Chamiec, Beata Zaborska, Maciej Tysarowski, Andrzej Budaj

**Affiliations:** 1 Department of Cardiology, Centre of Postgraduate Medical Education, Grochowski Hospital, Warsaw, Poland; 2 Department of Cardiovascular Medicine, Hartford Hospital, University of Connecticut School of Medicine, Hartford, CT, United States of America; Ospedale del Cuore G Pasquinucci Fondazione Toscana Gabriele Monasterio di Massa, ITALY

## Abstract

Exercise intolerance after acute myocardial infarction (AMI) is a predictor of worse prognosis, but its causes are complex and poorly studied. This study assessed the determinants of exercise intolerance using combined stress echocardiography and cardiopulmonary exercise testing (CPET-SE) in patients treated for AMI. We prospectively enrolled patients with left ventricular ejection fraction (LV EF) ≥40% for more than 4 weeks after the first AMI. Stroke volume, heart rate, and arteriovenous oxygen difference (A-VO_2_Diff) were assessed during symptom-limited CPET-SE. Patients were divided into four groups according to the percentage of predicted oxygen uptake (VO_2_) (Group 1, <50%; Group 2, 50–74%; Group 3, 75–99%; and Group 4, ≥100%). Among 81 patients (70% male, mean age 58 ± 11 years, 47% ST-segment elevation AMI) mean peak VO_2_ was 19.5 ± 5.4 mL/kg/min. A better exercise capacity was related to a higher percent predicted heart rate (Group 2 *vs*. Group 4, *p* <0.01), higher peak A-VO_2_Diff (Group 1 *vs*. Group 3, *p* <0.01) but without differences in stroke volume. Peak VO_2_ and percent predicted VO_2_ had a significant positive correlation with percent predicted heart rate at peak exercise (r = 0.28, *p* = 0.01 and r = 0.46, *p* < 0.001) and peak A-VO_2_Diff (r = 0.68, *p* <0.001 and r = 0.36, *p* = 0.001) but not with peak stroke volume. Exercise capacity in patients treated for AMI with LV EF ≥40% is related to heart rate response during exercise and peak peripheral oxygen extraction. CPET-SE enables non-invasive assessment of the mechanisms of exercise intolerance.

## Introduction

Exercise intolerance after acute myocardial infarction (AMI) is common and it indicates a poor prognosis [1–5]. In a previous study of 2,896 patients with newly diagnosed ischemic heart disease, which included 1,064 patients post-AMI, exercise capacity assessed before cardiac rehabilitation was significantly decreased at roughly 60% of age-matched values of healthy individuals without heart disease [3]. Contributors of low exercise capacity after AMI are complex and can include cardiac ischemic injury, systolic and diastolic dysfunction, functional mitral regurgitation, chronotropic incompetence, as well as peripheral muscle dysfunction [6, 7]. Deconditioning during the recovery period after AMI can result in changes within the skeletal muscles, similar to those observed in chronic heart failure [8]. Resting left ventricular function parameters, including left ventricular ejection fraction (LV EF), poorly correlate with exercise capacity; therefore, other mechanisms, such as peripheral factors or left ventricular function during exercise, need to be investigated [7, 9–11]. According to Fick’s equation, parameters contributing to oxygen uptake (VO_2_) are stroke volume, heart rate, and arteriovenous oxygen difference (A-VO_2_Diff) [12]. The contribution of each of these factors to VO_2_ varies depending on the individual patient’s disease profile; however, they have not been investigated in patients treated for AMI. In patients with preserved left ventricular systolic function, peripheral mechanisms, such as oxygen consumption by working muscles, may play a significant role in exercise limitation. Major factors contributing to A-VO_2_Diff are oxygen delivery to working muscles, capillary density, oxygen diffusion to mitochondria, and muscle aerobic capacity [13]. Although A-VO_2_Diff can be measured invasively, non-invasive assessment may allow wider applications in daily clinical practice.

Simultaneously performed stress echocardiography and cardiopulmonary exercise testing (CPET-SE) enables noninvasive assessment of cardiac and pulmonary function, as well as peripheral oxygen extraction. It is an emerging diagnostic method with considerable potential in cardiology, particularly in evaluation of the predictors of exercise intolerance [14–20]. It has mainly been used in studies of patients with heart failure [14, 15, 17, 19, 20]. Recently, it has also been applied to patients at risk of developing heart failure [21, 22].

The present study assessed the determinants of exercise capacity using CPET-SE in patients treated for AMI with LV EF ≥40%. Our findings indicate that CPET-SE enables non-invasive assessment of the mechanisms of exercise intolerance.

## Materials and methods

### Study sample

We prospectively enrolled consecutive patients aged >18 years who underwent percutaneous coronary intervention for their first AMI, between October 2015 and January 2019, at our cardiology department.

**Fig 1:**
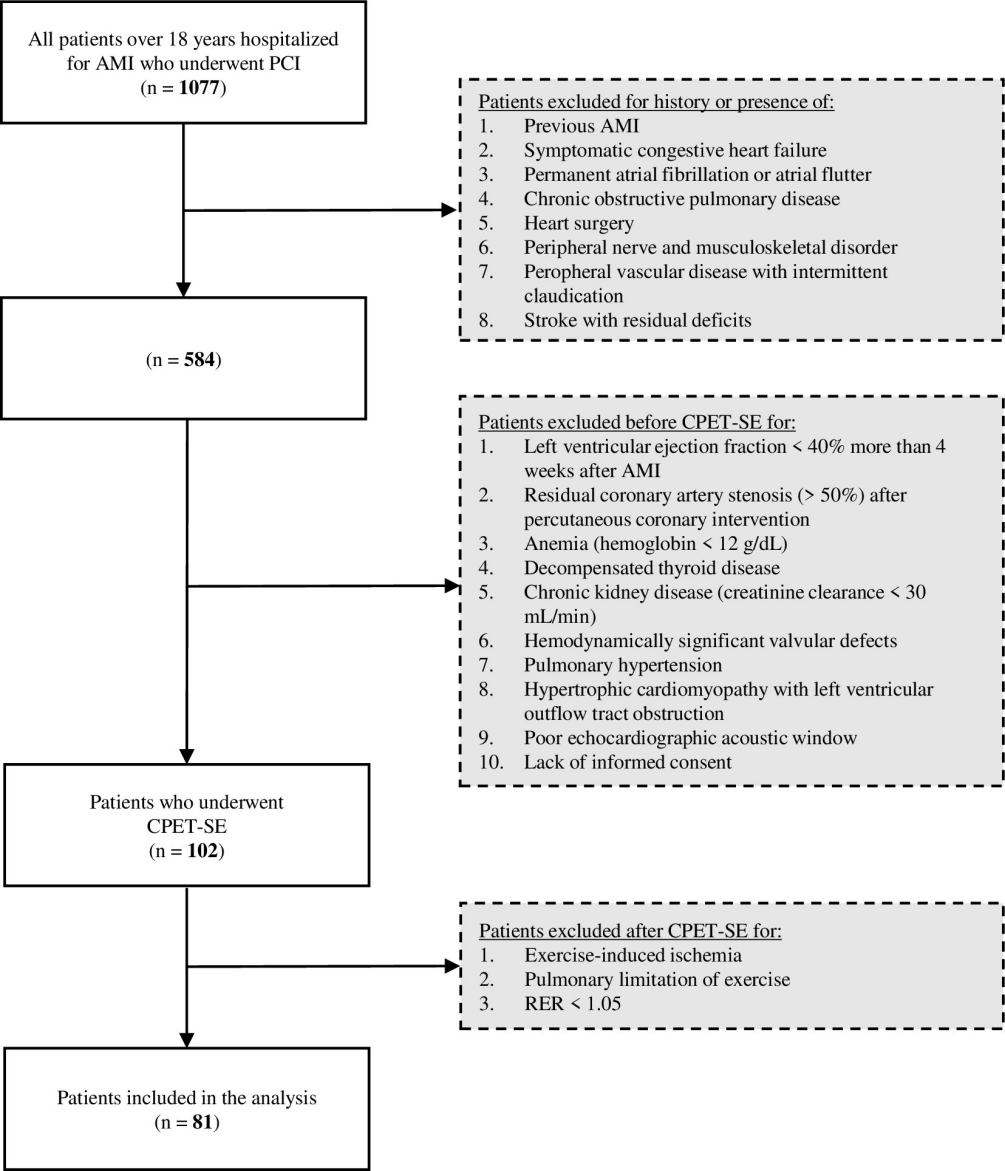
Study flow chart. Abbreviations: AMI, acute myocardial infarction; CPET-SE, combined stress echocardiography and cardiopulmonary exercise testing, FEV 1, forced expiratory volume in the first second; IVC, inspiratory vital capacity; PCI, percutaneous coronary intervention; RER, respiratory exchange ratio.

The flow chart of the study is presented in Fig 1. The study exclusion criteria were: previous AMI, history or presence of symptomatic congestive heart failure, permanent atrial fibrillation or atrial flutter, chronic obstructive pulmonary disease, heart surgery, peripheral nerve or musculoskeletal disorder, peripheral vascular disease with intermittent claudication, stroke with residual deficits, LV EF <40% at least 4 weeks after AMI, residual coronary artery stenosis (>50%) after percutaneous coronary intervention, anemia (hemoglobin <12 g/dL), decompensated thyroid disease, chronic kidney disease (creatinine clearance <30 mL/min), severe valvular diseases, pulmonary hypertension, hypertrophic cardiomyopathy with left ventricular outflow tract obstruction, exercise-induced ischemia, pulmonary limitations of exercise (breathing reserve at peak exercise <15%), respiratory exchange ratio (RER) at peak exercise <1.05, poor echocardiographic acoustic window, and lack of informed consent.

We collected data on demographic characteristics, medical history, and treatments as baseline characteristics. Self-assessed physical activity prior to AMI was categorized as low, moderate, or high according to the International Physical Activity Questionnaire [23].

### Cardiopulmonary exercise testing

We performed symptom-limited cardiopulmonary exercise tests using a Schiller Cardiovit CS-200 (Schiller, Baar, Switzerland) and an Ergo Spiro adapter (Ganshorn, Niederlauer, Germany) with patients on a semi-supine cycle ergometer eBike EL (Ergoline GmbH, Bitz, Germany). Volumetric and gas calibration was performed daily before the tests. Volumetric calibration for current temperature, relative air humidity, and atmospheric pressure was performed with a standard 2-L syringe. Gas calibration was performed using a standard gas mixture containing 15% oxygen, 6% carbon dioxide, and 79% nitrogen. In all cases, we used a ramp protocol with an incremental load of 12.5 watts/min. All patients were familiar with the exercise protocol and were encouraged to exercise at maximal effort (≥8 points using the 10-point Borg scale) [24]. All exercise tests were supervised and analyzed according to current guidelines [25–28]. During the stress test, we assessed the clinical and hemodynamic status of the patient, recorded 12-lead electrocardiograms, and recorded ventilation and gas exchange parameters. The peak VO_2_ was averaged from the highest 20 s of exercise, in mL/kg/min. Maximum predicted VO_2_ was calculated according to the Wasserman/Hansen equations [29]. The anaerobic threshold was calculated using a dual method approach (V-slope and ventilatory equivalent methods). Other analyzed cardiopulmonary exercise testing parameters included oxygen uptake to work rate increment ratio (ΔO_2_/ΔWR), ventilatory efficiency (VE/VCO_2_ slope), and breathing reserve at peak exercise, calculated as the percentage of maximum voluntary ventilation: [(maximum voluntary ventilation − minute ventilation at peak exercise) / maximum voluntary ventilation] × 100. The systolic and diastolic blood pressure and heart rate were recorded at rest and at peak exercise during the test, and the chronotropic index and percentage of maximum predicted heart rate at peak exercise was calculated [27]. The maximum predicted heart rate was calculated as 220–age in years [30]. Heart rate recovery was calculated as the difference between peak heart rate and heart rate at the first minute of recovery. Recorded electrocardiographic parameters included the presence or absence of ischemic changes, arrhythmias and conduction abnormalities according to the American Heart Association [27].

### Stress echocardiography

Echocardiography was performed at rest and at peak exercise using a VIVID 9 ultrasound machine (General Electric Medical System, Horten, Norway). Exercise echocardiography was carried out simultaneously with cardiopulmonary exercise testing. Resting echocardiograms were recorded in semi-recumbent position before starting the exercise. Exercise echocardiographic images were recorded at peak exercise, before effort termination. Two-dimensional images were recorded in standard views. Left ventricular volumes were measured in 4- and 2- chamber apical views and LV EF was calculated using the modified Simpson’s rule [31]. Left ventricular systolic (s’) and early diastolic (e’) myocardial velocities were evaluated using pulsed-tissue Doppler at the basal segments of the interventricular septum and lateral wall and were presented as averaged values. Regional wall motion was assessed and graded using a 4-point scale, where 1 represented normal and 4 represented dyskinetic motion in a 16-segment model and was expressed as a wall motion score index (WMSI). Mitral flow was assessed as early mitral inflow velocity (E), late (atrial) inflow velocity (A), and deceleration time using a pulse-wave Doppler sample volume between the mitral leaflet tips [32]. In cases with E to A fusion at peak exercise, diastolic function was assessed in the early recovery phase. Stroke volume was calculated by multiplying the area of the left ventricular outflow tract at rest by the left ventricular outflow tract velocity-time integral (in pulsed-wave Doppler averaged from three cardiac cycles at rest and at peak exercise). Right ventricular systolic function was assessed by evaluating tricuspid annular plane systolic excursion (TAPSE) and right ventricular systolic myocardial velocity (RV s’) in the 4-chamber apical view. The A-VO_2_Diff was calculated using the Fick equation as follows: VO_2_/cardiac output calculated from echocardiography [12, 14, 15]. Measurements and recordings of echocardiographic parameters were performed according to the American Society of Echocardiography and the European Association of Echocardiography recommendations [31–34]. Images were analyzed off-line using EchoPAC PC software v.110.0.x (GE Healthcare).

All CPET-SE examinations were performed and interpreted by one cardiologist experienced in stress echocardiography and cardiopulmonary exercise testing.

### Statistical analyses

Data are presented as mean (± standard deviation) or median (IQR) for continuous variables. Categorical variables are presented as numbers (percentages). Normality for all continuous variables was tested using the Shapiro–Wilk test. Group comparisons between continuous variables were performed using Welch’s *t*-test or Mann–Whitney U test, and the Fisher exact test or χ2 (chi-squared) test for categorical variables. Analysis of variance was used for multiple group comparisons of normally distributed numeric data.

Parameters associated with exercise capacity were compared among four groups, defined by the percentage of predicted VO_2_ (Group 1, <50%; Group 2, 50–74%; Group 3, 75–99%; and Group 4, ≥100%) [26, 28], using analysis of variance with the Bonferroni correction (Bonferroni post-hoc test). All statistical analyses were performed using R Statistical Software version 3.6.1, R Foundation for Statistical Computing, Vienna, Austria.

### Ethical statement

This study was conducted in conformance with the requirements set out in the Declaration of Helsinki. All patients provided written informed consent to participate. The study and all its protocols were approved by the Institutional Ethics Committee of the Centre of Postgraduate Medical Education Bioethical Committee (protocol code 16/PB/2015, approved on February 25, 2015).

## Results

### Baseline characteristics

Of 102 eligible patients, 81 patients (57 male, mean age 58 ± 11 years) who had undergone after CPET-SE were enrolled into analysis. Among these, 47% had AMI with and 53% had AMI without ST segment elevation. The included patients were mostly in Killip class 1. Only 3 patients in the group 3 were in Killip class 2. Patients in Group 4 were mostly women (*p* = 0.045 for Group 2 *vs*. Group 4) and had lower BMI (*p* = 0.011 for Group 2 *vs*. Group 4). Clinical characteristics are presented in Table 1. Almost 31% of the study sample had diabetes mellitus or impaired glucose tolerance, 67% had hypertension, and 47% were smokers. All patients were on optimal medical therapy for AMI, with 70 patients on beta-blockers. Beta-blockers were not withheld before the exercise tests.

**Table 1:** Demographic and clinical characteristics during hospitalization for acute myocardial infarction according to percentage of predicted oxygen uptake (Group 1, <50%; Group 2, 50–74%; Group 3, 75–99%; and Group 4, ≥100%).

Variables	Total		Group 1	Group 2	Group 3		Group 4	p*
	(n = 81)		(n = 7)	(n = 41)	(n = 24)		(n = 9)	
**Demographics**								
Male sex, n (%)	57 (70)		6 (86)	31 (76)	18 (75)		2 (22)	0.045^a^
Age, years	57.6 ± 11.0		58.8 ± 13.6	56.8 ± 9.4	57.1 ± 12.9		61.8 ± 10.9	0.606
Body mass index, kg/m^2^	28 ± 4		29 ± 5	28 ± 4	27 ± 4		24 ± 3	0.011^b^
**Comorbidity, n (%)**								
Current smoking	38 (47)		5 (71)	25 (61)	6 (25)		2 (22)	0.066
Hypertension	54 (67)		5 (71)	27 (66)	15 (62)		7 (78)	1
Hyperlipidemia	63 (78)		6 (86)	34 (83)	16 (67)		7 (78)	1
Diabetes mellitus/Impaired glucose tolerance	25 (31)		0 (0)	13 (32)	8 (33)		4 (44)	0.874
Paroxysmal atrial fibrillation	2 (2)		0 (0)	1 (2)	1 (4)		0 (0)	1
Physical activity before myocardial infarction								
Low	17 (21)		4 (57)	6 (15)	6 (25)		1 (11)	0.239
Moderate	41 (51)		3 (43)	24 (59)	10 (42)		4 (44)	1
High	23 (28)		0 (0)	11 (27)	8 (33)		4 (44)	0.874
**Clinical characteristics**								
STEMI, n (%)	38 (47)		3 (43)	21 (51)	11 (46)		3 (33)	1
Non STEMI, n (%)	43 (53)		4 (57)	20 (49)	13 (54)		6 (67)	1
Culprit lesion, n (%)								
Right coronary artery	20 (25)		1 (14)	13 (32)	4 (17)		2 (22)	1
Left anterior descending artery	47 (58)		2 (28)	22 (54)	12 (50)		7 (78)	1
Circumflex artery	19 (23)		5 (71)	6 (15)	6 (25)		2 (22)	1
Troponin T the highest value, ng/L, median (IQR)	784 (242, 2216)		2380 (571, 4107)	784 (204, 3225)	859 (450, 1777)		242 (162, 828)	1
Hemoglobin at discharge, g/dL	14.05 ± 1.21		14.19 ± 1.28	14.21 ±1.21	13.95 ± 1.14		13.50 ± 1.40	0.386
Creatinine clearance at discharge, mL/min	108 ± 30		113 ± 52	113 ± 28	104 ± 24		92 ± 29	0.245
LV EF at discharge, %, median (IQR)	55 (50, 60)		60 (55, 60)	52 (46, 57)	55 (50, 58)		60 (60, 60)	0.380
**Medication at discharge,** n (%)								
ACE-I/ARB	78 (96)		7 (100)	39 (95)	23 (96)		9 (100)	1
Beta-blocker	70 (86)		6 (86)	36 (88)	20 (83)		8 (89)	1
Aspirin	80 (99)		7 (100)	40 (98)	24 (100)		9 (100)	1
P2Y_12_ inhibitors	79 (97)		7 (100)	39 (95)	24 (100)		9 (100)	1
Statin	79 (98)		7 (100)	40 (98)	23 (96)		9 (100)	1
Nitrate	4 (5)		0 (0)	1 (2)	2 (8)		1 (11)	1
Calcium channel blocker	19 (23)		4 (57)	8 (20)	5 (21)		2 (22)	0.590
Diuretic	22 (27)		2 (29)	12 (29)	5 (21)		3 (33)	1

Note: Values represent mean ± SD, median (IQR) or number (%). P2Y_12_ inhibitors: clopidogrel or ticagrelor. Creatinine clearance was calculated according to Cockroft-Gault equation.

Abbreviations: ACE-I, angiotensin converting enzyme inhibitors; ARB, angiotensin receptor blockers; CPET-SE, combined stress echocardiography and cardiopulmonary exercise testing; LV EF, left ventricular ejection fraction; STEMI, acute myocardial infarction with ST segment elevation.

Bonferroni post hoc test (by Group) for significant *p* value:

^a^Group 2 *vs*. Group 4

^b^Group 2 *vs*. Group 3

* for non-significant presented the lowest *p* value.

### Combined stress echocardiography and cardiopulmonary exercise testing

Cardiopulmonary parameters in groups according to the percentage of predicted VO_2_ are presented in Table 2. The median (IQR) time from AMI to CPET-SE was 42 (32–53) days without significant differences between groups. The mean peak VO_2_ was 19.5 ± 5.4 mL/kg/min (20.6 ± 4.8 mL/kg/min for men and 16.8 ± 5.8 for women). The median (IQR) RER at peak exercise was 1.14 (1.07–1.21). Mean breathing reserve at peak exercise was 53 ± 12%, and no participants had a breathing reserve of <15% at peak exercise. The heart rate response to exercise (percent predicted heart rate) was the highest in Group 4 (*p* <0.01 for Group 2 *vs*. Group 4). The ΔO_2_/ΔWR was the lowest in Group 1 (*p* <0.001 for Group 1 *vs*. Group 3 and Group 4).

**Table 2:** Cardiopulmonary parameters during combined exercise testing according to percentage of predicted oxygen uptake (Group 1, <50%; Group 2, 50–74%; Group 3, 75–99%; and Group 4, ≥100%).

Variables	Total	Group 1	Group 2	Group 3	Group 4	p*
	(n = 81)	(n = 7)	(n = 41)	(n = 24)	(n = 9)	
Time from AMI to CPET-SE, days, median (IQR)	46 (36, 75)	40 (35, 44)	48 (39, 74)	45 (34, 82)	51 (44, 77)	0.321
Exercise time, sec	423 ± 143	347 ± 167	416 ± 132	478 ± 146	366 ± 131	0.132
Load peak, watts	102 ± 30	84 ± 35	100 ± 28	114 ± 31	90 ± 28	0.092
VO_2_-AT, mL/kg/min	12.1 ± 3.1	8.1 ± 1.6	11.2 ± 2.6	13.6 ± 2.8	15.4 ± 1.5	< 0.001^a^
Peak VO_2_ mL/kg/min	19.5 ± 5.4	11.9 ± 3.0	17.8 ± 3.9	22.8 ± 5.1	24.5 ± 3.7	< 0.001^a^
Percent predicted VO_2_, %, median (IQR)	72 (61, 83)	42 (40, 48)	66 (59, 71)	83 (77, 91)	108 (105, 110)	< 0.001^b^
ΔO_2_/ΔWR, mL/min/watt	12.38 ± 2.22	9.47 ± 2.57	11.85 ± 1.58	13.58 ± 2.10	13.83 ± 1.92	< 0.01^c^
						<0.001^d^
VCO_2_ peak, L/min	1.83 ± 0.59	1.41 ± 0.48	1.75 ± 0.56	2.09 ± 0.61	1.88 ± 0.52	0.027^e^
RER peak, median (IQR)	1.12 (1.07, 1.21)	1.30 (1.18, 1.31)	1.11 (1.06, 1.20)	1.12 (1.07, 1.20)	1.15 (1.12, 1.21)	0.028^f^
Heart rate rest, bpm	68 ± 9	70 ± 16	68 ± 8	65 ± 8	73 ± 7	0.161
Heart rate peak, bpm, median (IQR)	109 (102, 116)	104 (95, 109)	107 (98, 113)	112 (107, 115)	116 (111, 128)	0.097
Percent predicted heart rate, %, median (IQR)	67 (63, 72)	66 (59, 67)	65 (62, 70)	68 (65, 72)	76 (68, 79)	<0.01^g^
Chronotropic index, %	45 ± 14	40 ± 13	41 ± 15	49 ± 13	55 ± 13	0.031^g^
Heart rate recovery, bpm	18 ± 10	18 ± 12	17 ± 11	20 ± 7	23 ± 7	0.392
SBP rest, mmHg, median (IQR)	130 (120, 140)	130 (125, 135)	125 (120, 140)	127 (119, 140)	130 (120, 135)	0.926
DBP rest, mmHg, median (IQR)	75 (70, 80)	80 (70, 85)	79 (70, 80)	70 (70, 80)	80 (70, 80)	0.639
SBP peak, mmHg	181 ± 21	179 ± 25	182 ± 17	181 ± 27	182 ± 24	0.986
DBP peak, mmHg, median (IQR)	70 (60, 75)	70 (67, 72)	70 (65, 75)	65 (60, 75)	70 (60, 70)	0.475
VD/VT peak, median (IQR)	0.17 (0.14, 0.22)	0.17 (0.16, 0.22)	0.17 (0.14, 0.22)	0.16 (0.12, 0.19)	0.20 (0.15, 0.23)	0.810
VE/VCO_2_ slope	24 ± 5	27 ± 6	24 ± 5	23 ± 3	22 ± 3	0.176
Percent predicted IVC, %	75 ± 13	77 ± 10	77 ± 14	71 ± 14	73 ± 13	0.359
Percent predicted FEV1, %, median (IQR)	90 (83, 100)	96 (84, 97)	88 (84, 98)	94 (85, 109)	86 (78, 100)	0.412
FEV1/IVC, %, median (IQR)	90 (82, 99)	89 (83, 89)	87 (82, 96)	94 (86, 111)	90 (72, 99)	0.154

Note: Values represent mean ± SD, median (IQR) or number (%).

Abbreviations: AT, anaerobic threshold; DBP, diastolic blood pressure, FEV 1, forced expiratory volume in the first second; IVC, inspiratory vital capacity; RER, respiratory exchange ratio; SBP, systolic blood pressure; VCO_2_, carbon dioxide production; VD/VT, physiological dead space to tidal volume ratio; VE/VCO_2_ slope, minute ventilation to carbon dioxide production slope; VO_2_, oxygen uptake; ΔO_2_/ΔWR, oxygen uptake to work rate increment.

Bonferroni post hoc test (by Group) for significant *p* value:

^a^Group 3 *vs*. Group 4

^b^all Groups

^c^Group 1 *vs*. Group 2 and the Group 3 *vs*. Group 4

^d^Group 1 *vs*. Group 3; and Group 1 *vs*. Group 4, *p* = 0.026 for the Group 2 *vs* Group 4

^e^Group 1 *vs*. Group3

^**f**^Group 1 *vs*. Group 2; Group 1 *vs*. Group 3

^**g**^Group 2 *vs*. Group 4

* for non-significant presented the lowest *p* value.

The peak A-VO_2_Diff was lowest in Group 1 (*p* <0.01 for Group 1 *vs*. Group 3). Mild mitral regurgitation at peak exercise was most frequent in Group 3. No other significant differences in resting and stress echocardiography parameters were noticed (Table 3).

**Table 3:** Stress echocardiography parameters during combined exercise testing according to percentage of predicted oxygen uptake (Group 1, <50%; Group 2, 50–74%; Group 3, 75–99%; and Group 4, ≥100%).

Variables	Total	Group 1	Group 2	Group 3	Group 4	p*
	(n = 81)	(n = 7)	(n = 41)	(n = 24)	(n = 9)	
**Rest**						
LVOT diameter, cm	2.13 ± 0.16	2.17 ± 0.08	2.16 ± 0.17	2.10 ± 0.15	2.04 ± 0.15	0.194
LVOT VTI, cm, median (IQR)	21.5 (20.0, 23.2)	21.1 (20.5, 23.0)	21.5 (19.9, 23.5)	21.4 (20.7, 23.2)	22.4 (21.9, 23.0)	0.708
Stroke volume, mL, median (IQR)	75 (68, 87)	77 (74, 87)	76 (68, 89)	72 (67, 86)	75 (70, 82)	0.611
WMSI, median (IQR)	1.19 (1.06, 1.31)	1.06 (1.06, 1.22)	1.25 (1.06, 1.38)	1.16 (1.06, 1.20)	1.06 (1.06, 1.12)	0.089
LV EF, %	57 ± 8	63 ± 13	57 ± 7	56 ± 6	57 ± 4	0.127
LV EDV, mL	100.2 ± 30.1	111.1 ± 37.9	101.2 ± 31.5	103.7 ± 26.5	77.6 ± 15.9	0.117
LV ESV, mL	43.5 ± 17.7	44.1 ± 29.6	44.1 ± 17.9	46.0 ± 15.6	33.4 ± 7.3	0.274
E/A ratio, median (IQR)	0.96 (0.73, 1.20)	0.82 (0.70, 1.32)	0.98 (0.73, 1.20)	0.99 (0.79, 1.18)	0.81 (0.73, 1.09)	0.902
Deceleration time, ms, median (IQR)	243 (201, 269)	243 (206, 261)	242 (208, 269)	245 (203, 278)	232 (191, 254)	0.894
TAPSE, cm	2.24 ± 0.27	2.27 ± 0.29	2.26 ± 0.30	2.20 ± 0.27	2.21 ± 0.17	0.819
RV s’, cm/s, median (IQR)	12 (11, 14)	11 (10, 13)	12 (11, 13)	12 (11, 13)	12 (11, 15)	0.804
LV s’, cm/s, median (IQR)	7 (7, 9)	9 (7, 11)	8 (7, 8)	7 (7, 8)	7 (7, 8)	0.130
e’, cm/s	9 ± 2	10 ± 3	9 ± 2	9 ± 2	9 ± 2	0.565
E/e’ ratio, median (IQR)	8 (6, 9)	7 (6, 8)	8 (6, 9)	7 (6, 9)	9 (7, 12)	0.186
Mitral regurgitation mild, n (%)	48 (59)	4 (57)	20 (49)	18 (75)	6 (67)	0.422
Mitral regurgitation moderate, n (%)	2 (2)	1 (14)	1 (2)	0 (0)	0 (0)	1
Tricuspid regurgitation mild, n (%)	30 (37)	1 (14)	15 (37)	12 (50)	2 (22)	1
Tricuspid regurgitation moderate, n (%)	0 (0)	0 (0)	0 (0)	0 (0)	0 (0)	-
A-VO_2_Diff, mL/dL	8 ± 3	7 ± 3	8 ± 3	8 ± 2	8 ± 2	0.769
**Peak exercise**						
LVOT VTI, cm	27.1 ± 4.1	25.7 ± 4.2	26.7 ± 4.0	28.2 ± 4.3	27.0 ± 4.2	0.504
Stroke volume, mL, median (IQR)	93 (82, 110)	92 (88, 96)	97 (83, 114)	95 (84, 109)	88 (74, 96)	0.561
WMSI, median (IQR)	1.12 (1.06, 1.25)	1.06 (1.06, 1.22)	1.19 (1.06, 1.31)	1.09 (1.06, 1.19)	1.06 (1.06, 1.06)	0.056
LV EF, %	66 ± 9	72 ± 13	65 ± 8	66 ± 10	70 ± 7	0.270
LV EDV, mL	97.3 ± 28.6	96.8 ± 30.4	102.3 ± 31.9	97.0 ± 22.0	75.8 ± 19.6	0.059
LV ESV, mL	34.0 ± 16.1	29.4 ± 21.1	37.2 ± 16.5	34.1 ± 14.9	22.7 ± 7.9	0.068
E/A ratio	1.28 ± 0.45	1.21 ± 0.52	1.28 ± 0.50	1.35 ± 0.38	1.16 ± 0.35	0.694
Deceleration time, ms, median (IQR)	172 (153, 206)	190 (175, 250)	169 (153, 207)	169 (149, 178)	187 (161, 207)	0.659
TAPSE, cm	2.91 ± 0.51	2.84 ± 0.54	2.89 ± 0.57	3.02 ± 0.42	2.74 ± 0.37	0.484
RV s’, cm/s	16 ± 3	16 ± 3	16 ± 3	17 ± 3	16 ± 2	0.081
LV s’, cm/s, median (IQR)	10 (9, 11)	10 (10, 11)	10 (9, 11)	10 (9, 12)	9 (9, 11)	0.332
e’, cm/s	12 ± 3	13 ± 3	12 ± 3	13 ± 2	12 ± 3	0.427
E/e’ ratio, median (IQR)	8 (7, 10)	7 (6, 8)	8 (7, 10)	8 (7, 10)	8 (8, 11)	0.390
Mitral regurgitation mild, n (%)	52 (64)	5 (71)	21 (51)	20 (83)	6 (67)	0.042^b^
Mitral regurgitation moderate, n (%)	7 (9)	1 (14)	4 (10)	1 (4)	1 (11)	1
Tricuspid regurgitation mild, n (%)	32 (39)	2 (29)	14 (34)	15 (62)	1 (11)	0.151
Tricuspid regurgitation moderate, n (%)	1 (1)	0 (0)	0 (0)	1 (4)	0 (0)	1
A-VO_2_Diff, mL/dL	15 ± 4	11 ± 4	15 ± 4	17 ± 5	16 ± 3	<0.01^a^

Note: Values represent mean ± SD, median (IQR) or number (%).

Abbreviations A, late mitral inflow velocity; A-VO2Diff, arteriovenous oxygen difference; E, early mitral inflow velocity; e’, early diastolic myocardial velocity; LV EF, left ventricular ejection fraction; LV EDV, left ventricular end-diastolic volume; LV ESV, left ventricular end-systolic volume; LV s’, left ventricular systolic myocardial velocity; LVOT VTI, left ventricular outflow tract velocity time integral; RV s’, right ventricular systolic myocardial velocity; TAPSE, tricuspid annulus plane systolic excursion; WMSI, wall motion score index.

Bonferroni post hoc test (by Group) for significant *p* value:

^a^Group 1 *vs*. Group 3

^b^Group 2 *vs*. Group 3

* for non-significant presented the lowest *p* value.

### Determinants of exercise capacity

**Fig 2:**
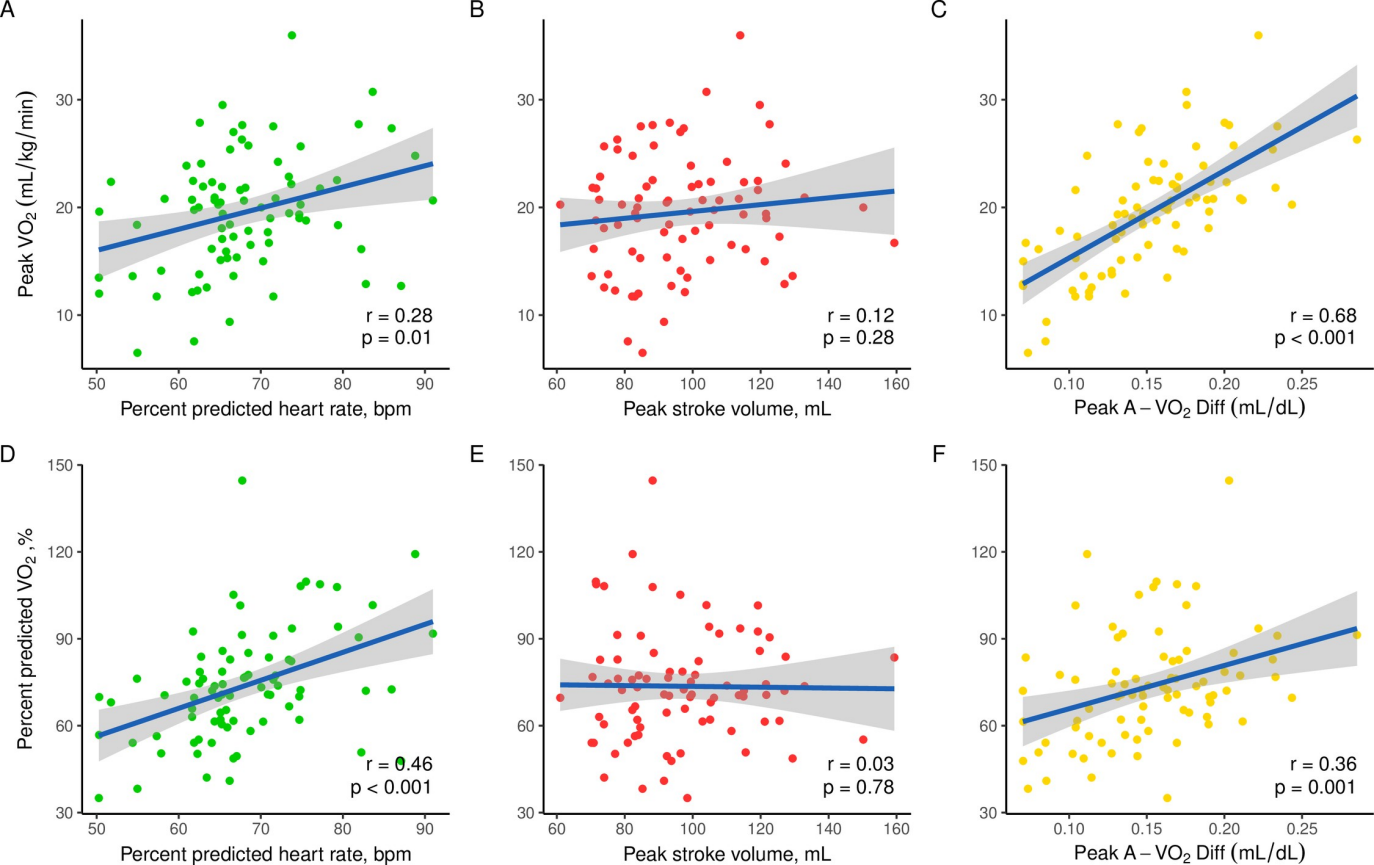
Linear regression between peak oxygen uptake, percent predicted oxygen uptake and percent predicted heart rate, peak stroke volume, and peak arteriovenous oxygen difference in the whole studied group. Abbreviations: A-VO_2_Diff, arteriovenous oxygen difference; VO_2_, oxygen uptake.

Peak VO_2_ and percent predicted VO_2_ had a significant positive correlation with percent predicted heart rate at peak exercise (r = 0.28, *p* = 0.01 and r = 0.46, *p* < 0.001) and peak A-VO_2_Diff (r = 0.68, *p* <0.001 and r = 0.36, *p* = 0.001) but not with peak stroke volume (Fig 2).

Spearman correlations for peak VO_2_, percent predicted VO_2_ and clinical and CPET-SE data are presented in S1 Table. Moderately to strong correlations were found for peak VO_2_ and heart rate at peak exercise, chronotropic index, ΔO_2_/ΔWR, VE/VCO_2_ slope, A-VO_2_Diff, and LV s’ at peak exercise and also for percent predicted VO_2_ and heart rate at peak exercise, percent predicted heart rate, chronotropic index, ΔO_2_/ΔWR, and A-VO_2_Diff at peak exercise.

## Discussion

Our study revealed that in post-AMI patients with LV EF ≥40%, exercise capacity is related to heart rate response during exercise and peak peripheral oxygen extraction. Impaired extraction of oxygen by working muscles plays a significant contribution in the most compromised patients.

Several causes of exercise intolerance, leading to low peak VO_2_, have been described; they include Fick variables, such as stroke volume, heart rate, A-VO_2_Diff, and non-Fick variables, such as motivation; pulmonary, central, and peripheral nervous system diseases; peripheral arterial or vein pathologies; or bone and joint abnormalities [35]. We excluded patients with non-Fick variables that led to exercise termination from our study. Heart rate and A-VO_2_Diff have the largest dynamic response during exercise. Both parameters can increase up to 2.5-fold, contrary to stroke volume, which increases up to 1.4-fold during maximal exercise [36]. Among these parameters, stroke volume and heart rate during exercise reflect cardiac function, while A-VO_2_Diff reflects peripheral oxygen extraction.

### Exercise capacity after acute myocardial infarction

The exercise capacity in our study group was similar to that of previous studies on post-AMI patients entering cardiac rehabilitation. In an observational study of 12,169 male rehabilitation candidates tested on a cycle ergometer, the mean peak VO_2_ was 20.5 ± 5.2 mL/kg/min [1]. In a similar study of 2,380 women, the mean peak VO_2_ was 15.4 ± 4.0 mL/kg/min [2]. Moreover, in another group of 2,896 patients who exercised on a treadmill after AMI and before cardiac rehabilitation, the mean peak VO_2_ was 20.4 ± 6.6 mL/kg/min and 14.7 ± 4.2 mL/kg/min in men and women, respectively [3]. These studies focused on the prognostic significance of exercise capacity, but not on the mechanisms leading to low peak VO_2_.

### Contributors to exercise intolerance

To the best of our knowledge, no previous study described mechanisms of exercise intolerance in patients treated for AMI with LV EF ≥ 40% using CPET-SE. Heart rate response and peripheral factors were previously found to be the main contributors to reduced exercise capacity, in healthy subjects and in patients with heart failure but with preserved LV EF (HFpEF) or midrange LV EF (HFmrEF). A small study of 14 subjects with normal cardiac function and 16 patients with HFpEF examined effort intolerance using CPET-SE, with noninvasively calculated A-VO_2_Diff. They found that heart rate and A-VO_2_Diff, but not stroke volume, at peak exercise were the most significant independent predictors of peak VO_2_. However, among patients with HFpEF, diastolic dysfunction was also found to be a determinant of peak VO_2_ [15].

In another study of 48 patients with HFpEF assessed with CPET-SE, both reduced cardiac output and calculated A-VO_2_Diff contributed significantly to exercise intolerance. In this study, the strongest independent predictor of peak VO_2_ was the change in A-VO_2_Diff from rest to peak exercise [14].

In a study of 169 subjects (healthy controls and heart failure patients with a wide range of LV EF) assessed with CPET-SE, where A-VO_2_Diff was calculated noninvasively based on Fick’s equation, multivariate analysis revealed that peak VO_2_ was predominantly influenced by peripheral factors (such as A-VO_2_Diff) in patients with HFmrEF and HFpEF, whereas it was influenced by decreased stroke volume in patients with reduced LV EF [19].

In another, recently published study involving patients with hypertension with and without HFpEF, reduced peak VO_2_ was found to be related to decreased calculated peak A-VO_2_Diff [20]. Like our study, a study of 278 patients with various degrees of heart failure showed that A-VO_2_Diff was a significant contributor to exercise capacity in the most compromised group (percent predicted VO_2_ <50%) [37].

Similar mechanisms of exercise intolerance were also found in a study with cardiopulmonary exercise testing with invasive hemodynamic monitoring. Directly measured peak A-VO_2_Diff has been found to be the major exercise-limiting factor in patients with HFpEF [38].

In our study, chronotropic response parameters, such as the percentage of predicted heart rate and the chronotropic index were highest in the group with the highest exercise capacity. Although the majority of our patients were on beta-blockers, we did not analyze beta-blocker doses. Our recent research suggested that exercise capacity is related to chronotropic response during exercise, rather than to the beta-blocker doses [39]. Aerobic training could improve chronotropic responses to exercise by adaptation of autonomic function [40], and could improve A-VO_2_Diff by enhancing endothelial function and skeletal muscle deoxygenation [41, 42].

Although previous studies have demonstrated the influence of diastolic dysfunction on exercise capacity [43, 44], our study did not support these findings. In our study, elevated E/e’ ratio >14 at peak exercise was recorded in patients with better exercise capacity (2 patients in Group 3, and 1 patient in Group 4). The lack of significant impact of diastolic function on exercise capacity could be related to the underrepresentation of such patients in our sample.

In patients treated for AMI, functional impairment may be caused by exercise-induced functional mitral regurgitation [45, 46]. In our studied group, no significant deterioration of mitral regurgitation was noticed during exercise. Mild mitral regurgitation during exercise was most frequent in Group 3, but only 7 patients had moderate, and none had severe mitral regurgitation.

### Combined stress echocardiography and cardiopulmonary exercise testing

The increased accessibility of CPET-SE provides the opportunity for noninvasive assessment of cardiac and peripheral factors of exercise intolerance. However, CPET-SE is not methodologically standardized. The use of a cycle ergometer in a semi-recumbent position has been suggested to offer improved echocardiographic evaluation [16, 18].

Combined use of stress echocardiography and cardiopulmonary exercise testing allows the identification of central (low peak stroke volume, chronotropic incompetence) or peripheral (low peak A-VO_2_Diff) mechanisms of exercise intolerance. Two representative patients from our study, patient A with reduced and patient B with good exercise capacity, are presented in S2 Table.

The combination of cardiopulmonary exercise testing with exercise stress echocardiography is a valuable diagnostic tool and its clinical utility has been proven in the diagnostic evaluation of many cardiac diseases, including heart failure; cardiomyopathies; pulmonary arterial hypertension; valvular heart disease, and coronary artery disease [14, 15, 19, 20, 47–49]. Furthermore, CPET-SE provides additional information in the case of patients who do not have heart failure, but have unexplained exercise dyspnea [50]. Exercise pulmonary hypertension due to mitral regurgitation or left ventricular dysfunction can also lead to effort intolerance [46, 48]. Furthermore, elevated left ventricular filling pressure during exercise in patients with exercise intolerance and without diastolic dysfunction at rest can be identified by CPET-SE [47]. Because resting LV EF is weakly correlated with exercise capacity, there is a need to clarify other parameters contributing to exercise performance, including left and right ventricular contractile reserve, interventricular dependence, diastolic function, left atrial function, as well as peripheral factors [46].

### Limitations

This study has several limitations. We only included patients who were able to exercise. The mode of exercise used, a cycle ergometer in a semi-recumbent position, could cause lower-extremity muscle fatigue, particularly in untrained patients, and can lead to lower peak VO_2_ values compared to treadmill or to supine cycle ergometer. Respiratory movements at peak exercise can cause difficulties in image acquisition, and due to the angle-dependency of Doppler-measured velocities, could lead to underestimation of the calculated stroke volume.

A particular feature of our study was the noninvasive assessment of cardiac and peripheral mechanisms of exercise limitation. Noninvasively assessed A-VO_2_Diff, as calculated using Fick’s equation, is strongly related to oxygen uptake and cardiac output and rather reflects “non-cardiac” contributors to oxygen uptake and should not be equated to invasively measured A-VO_2_Diff. However, this method has been used in previous non-invasive studies [15, 17, 19, 20, 37, 45], and calculated A-VO_2_Diff values were similar to those measured in invasive studies [38, 51].

Our study applies only to patients with LV EF ≥ 40%. In patients with reduced LV EF, parameters of heart injury and stroke volume could play a more significant role in exercise intolerance. Furthermore, we recruited a relatively small group of patients from a single center. Some patients did not consent to participate in the study, introducing selection bias.

## Conclusions

Patients who have been treated for AMI, even those without reduced LV EF, remain at a risk of developing symptomatic heart failure [52]. Identification of factors responsible for exercise intolerance is crucial for their evaluation and management. CPET-SE enables non-invasive assessment of the mechanisms of exercise intolerance. In such patients, the heart rate response during exercise and peak peripheral oxygen extraction have the most marked effect on exercise intolerance. Our findings can help in clinical decision-making and can guide therapy to improve exercise capacity. Further studies with a larger group of post-AMI patients with various degrees of left ventricular dysfunction and with directly measured A-VO_2_Diff are needed to confirm our findings.

## Supporting information

S1 Table Spearman correlations for peak oxygen uptake, percent predicted oxygen uptake and clinical, and combined stress echocardiography and cardiopulmonary exercise testing data. Abbreviations: A, late mitral inflow velocity; A-VO_2_Diff, arteriovenous oxygen difference; DBP, diastolic blood pressure; E, early mitral inflow velocity; e’, early diastolic myocardial velocity; LV EF, left ventricular ejection fraction; LV EDV, left ventricular end-diastolic volume; LV ESV, left ventricular end-systolic volume; LV s’, left ventricular systolic myocardial velocity; ΔO_2_/ΔWR, oxygen uptake to work rate increment; RV s’, right ventricular systolic myocardial velocity; SBP, systolic blood pressure; TAPSE, tricuspid annulus plane systolic excursion; VE/VCO_2_ slope, minute ventilation to carbon dioxide production slope; VO_2_, oxygen uptake; WMSI, wall motion score index.
(DOCX)

S2 Table Summary of the combined stress echocardiography and cardiopulmonary exercise testing parameters proposed for functional phenotyping on the basis of 2 patients from the study. Patient A is an obese man with low physical activity, bad peripheral oxygen extraction, and low exercise capacity; patient B is a normal weight woman with high levels of daily physical activity, good peripheral oxygen extraction, and good exercise capacity. Abbreviations: A-VO_2_Diff, arteriovenous oxygen difference; BMI, body mass index; DBP, diastolic blood pressure; LV EF, left ventricular ejection fraction; SBP, systolic blood pressure; VE/VCO_2_ slope, minute ventilation to carbon dioxide production slope; VO_2_, oxygen uptake.
(DOCX)

## References

[1] Kavanagh T, Mertens DJ, Hamm LF, Beyene J, Kennedy J, Corey P, et al. Prediction of long-term prognosis in 12 169 men referred for cardiac rehabilitation. Circulation. 2002;106(6):666–71. Epub 2002/08/07. doi: 10.1161/01.cir.0000024413.15949.ed . 12163425

[2] Kavanagh T, Mertens DJ, Hamm LF, Beyene J, Kennedy J, Corey P, et al. Peak oxygen intake and cardiac mortality in women referred for cardiac rehabilitation. J Am Coll Cardiol. 2003;42(12):2139–43. Epub 2003/12/19. doi: 10.1016/j.jacc.2003.07.028 . 14680741

[3] Ades PA, Savage PD, Brawner CA, Lyon CE, Ehrman JK, Bunn JY, et al. Aerobic capacity in patients entering cardiac rehabilitation. Circulation. 2006;113(23):2706–12. Epub 2006/06/07. doi: 10.1161/CIRCULATIONAHA.105.606624 . 16754799

[4] Keteyian SJ, Brawner CA, Savage PD, Ehrman JK, Schairer J, Divine G, et al. Peak aerobic capacity predicts prognosis in patients with coronary heart disease. Am Heart J. 2008;156(2):292–300. Epub 2008/07/29. doi: 10.1016/j.ahj.2008.03.017 . 18657659

[5] Knuuti J, Wijns W, Saraste A, Capodanno D, Barbato E, Funck-Brentano C, et al. 2019 ESC Guidelines for the diagnosis and management of chronic coronary syndromes. Eur Heart J. 2020;41(3):407–77. Epub 2019/09/11. doi: 10.1093/eurheartj/ehz425 . 31504439

[6] Kaminsky LA, Arena R, Ellingsen Ø, Harber MP, Myers J, Ozemek C, et al. Cardiorespiratory fitness and cardiovascular disease—The past, present, and future. Progress in Cardiovascular Diseases. 2019;62(2):86–93. doi: 10.1016/j.pcad.2019.01.002 30639135

[7] Kaminsky LA, Myers J, Arena R. Determining Cardiorespiratory Fitness With Precision: Compendium of Findings From the FRIEND Registry. Prog Cardiovasc Dis. 2019;62(1):76–82. Epub 2018/11/06. doi: 10.1016/j.pcad.2018.10.003 . 30385268

[8] Warburton DE, Taylor A, Bredin SS, Esch BT, Scott JM, Haykowsky MJ. Central haemodynamics and peripheral muscle function during exercise in patients with chronic heart failure. Appl Physiol Nutr Metab. 2007;32(2):318–31. Epub 2007/05/09. doi: 10.1139/h06-085 . 17486176

[9] Franciosa JA, Park M, Levine TB. Lack of correlation between exercise capacity and indexes of resting left ventricular performance in heart failure. Am J Cardiol. 1981;47(1):33–9. Epub 1981/01/01. doi: 10.1016/0002-9149(81)90286-1 . 7457405

[10] Arena R, Myers J, Williams MA, Gulati M, Kligfield P, Balady GJ, et al. Assessment of functional capacity in clinical and research settings: a scientific statement from the American Heart Association Committee on Exercise, Rehabilitation, and Prevention of the Council on Clinical Cardiology and the Council on Cardiovascular Nursing. Circulation. 2007;116(3):329–43. Epub 2007/06/20. doi: 10.1161/CIRCULATIONAHA.106.184461 . 17576872

[11] Lancellotti P, Pellikka PA, Budts W, Chaudhry FA, Donal E, Dulgheru R, et al. The clinical use of stress echocardiography in non-ischaemic heart disease: recommendations from the European Association of Cardiovascular Imaging and the American Society of Echocardiography. Eur Heart J Cardiovasc Imaging. 2016;17(11):1191–229. Epub 2016/11/24. doi: 10.1093/ehjci/jew190 . 27880640

[12] Selzer A, Sudrann RB. Reliability of the determination of cardiac output in man by means of the Fick principle. Circ Res. 1958;6(4):485–90. Epub 1958/07/01. doi: 10.1161/01.res.6.4.485 . 13547408

[13] Poole DC, Hirai DM, Copp SW, Musch TI. Muscle oxygen transport and utilization in heart failure: implications for exercise (in)tolerance. American journal of physiology Heart and circulatory physiology. 2012;302(5):H1050–H63. Epub 2011/11/18. doi: 10.1152/ajpheart.00943.2011 . 22101528 PMC3311454

[14] Haykowsky MJ, Brubaker PH, John JM, Stewart KP, Morgan TM, Kitzman DW. Determinants of exercise intolerance in elderly heart failure patients with preserved ejection fraction. J Am Coll Cardiol. 2011;58(3):265–74. Epub 2011/07/09. doi: 10.1016/j.jacc.2011.02.055 ; PubMed Central PMCID: PMC3272542. 21737017 PMC3272542

[15] Shimiaie J, Sherez J, Aviram G, Megidish R, Viskin S, Halkin A, et al. Determinants of Effort Intolerance in Patients With Heart Failure: Combined Echocardiography and Cardiopulmonary Stress Protocol. JACC Heart Fail. 2015;3(10):803–14. Epub 2015/10/10. doi: 10.1016/j.jchf.2015.05.010 . 26449998

[16] Guazzi M, Bandera F, Ozemek C, Systrom D, Arena R. Cardiopulmonary Exercise Testing: What Is its Value? J Am Coll Cardiol. 2017;70(13):1618–36. Epub 2017/09/25. doi: 10.1016/j.jacc.2017.08.012 . 28935040

[17] Topilsky Y, Rozenbaum Z, Khoury S, Pressman GS, Gura Y, Sherez J, et al. Mechanisms of Effort Intolerance in Patients With Heart Failure and Borderline Ejection Fraction. Am J Cardiol. 2017;119(3):416–22. Epub 2016/11/27. doi: 10.1016/j.amjcard.2016.10.026 . 27887692

[18] Santoro C, Sorrentino R, Esposito R, Lembo M, Capone V, Rozza F, et al. Cardiopulmonary exercise testing and echocardiographic exam: an useful interaction. Cardiovasc Ultrasound. 2019;17(1):29. Epub 2019/12/05. doi: 10.1186/s12947-019-0180-0 ; PubMed Central PMCID: PMC6892222. 31796047 PMC6892222

[19] Pugliese NR, Fabiani I, Santini C, Rovai I, Pedrinelli R, Natali A, et al. Value of combined cardiopulmonary and echocardiography stress test to characterize the haemodynamic and metabolic responses of patients with heart failure and mid-range ejection fraction. Eur Heart J Cardiovasc Imaging. 2019;20(7):828–36. Epub 2019/02/13. doi: 10.1093/ehjci/jez014 . 30753369

[20] Pugliese NR, Mazzola M, Fabiani I, Gargani L, De Biase N, Pedrinelli R, et al. Haemodynamic and metabolic phenotyping of hypertensive patients with and without heart failure by combining cardiopulmonary and echocardiographic stress test. Eur J Heart Fail. 2020;22(3):458–68. Epub 2020/01/18. doi: 10.1002/ejhf.1739 . 31950651

[21] Pugliese NR, De Biase N, Gargani L, Mazzola M, Conte L, Fabiani I, et al. Predicting the transition to and progression of heart failure with preserved ejection fraction: a weighted risk score using bio-humoural, cardiopulmonary, and echocardiographic stress testing. Eur J Prev Cardiol. 2020. Epub 2021/02/25. doi: 10.1093/eurjpc/zwaa129 . 33624088

[22] Pugliese NR, De Biase N, Conte L, Gargani L, Mazzola M, Fabiani I, et al. Cardiac Reserve and Exercise Capacity: Insights from Combined Cardiopulmonary and Exercise Echocardiography Stress Testing. J Am Soc Echocardiogr. 2021;34(1):38–50. Epub 2020/10/11. doi: 10.1016/j.echo.2020.08.015 . 33036818

[23] Craig CL, Marshall AL, Sjostrom M, Bauman AE, Booth ML, Ainsworth BE, et al. International physical activity questionnaire: 12-country reliability and validity. Med Sci Sports Exerc. 2003;35(8):1381–95. Epub 2003/08/06. doi: 10.1249/01.MSS.0000078924.61453.FB . 12900694

[24] Borg G. Borg’s Perceived Exertion And Pain Scales. Champaign: Human Kinetics; 1998.

[25] ATS/ACCP Statement on cardiopulmonary exercise testing. Am J Respir Crit Care Med. 2003;167(2):211–77. Epub 2003/01/14. doi: 10.1164/rccm.167.2.211 . 12524257

[26] Guazzi M, Adams V, Conraads V, Halle M, Mezzani A, Vanhees L, et al. EACPR/AHA Scientific Statement. Clinical recommendations for cardiopulmonary exercise testing data assessment in specific patient populations. Circulation. 2012;126(18):2261–74. Epub 2012/09/07. doi: 10.1161/CIR.0b013e31826fb946 ; PubMed Central PMCID: PMC4777325. 22952317 PMC4777325

[27] Fletcher GF, Ades PA, Kligfield P, Arena R, Balady GJ, Bittner VA, et al. Exercise standards for testing and training: a scientific statement from the American Heart Association. Circulation. 2013;128(8):873–934. Epub 2013/07/24. doi: 10.1161/CIR.0b013e31829b5b44 . 23877260

[28] Guazzi M, Arena R, Halle M, Piepoli MF, Myers J, Lavie CJ. 2016 Focused Update: Clinical Recommendations for Cardiopulmonary Exercise Testing Data Assessment in Specific Patient Populations. Circulation. 2016;133(24):e694–711. Epub 2016/05/05. doi: 10.1161/CIR.0000000000000406 . 27143685

[29] Wasserman K, Hansen JE, Sue DY, Stringer WW, Sietsema KE, Sun X, et al. Normal values. Principles of exercise testing and interpretation including pathophysiology and clinical applications. 5th ed. Philadelphia: Wolters Kluwer/Lippincott Williams & Wilkins; 2012. p. 154–80.

[30] Astrand I. Aerobic work capacity in men and women with special reference to age. Acta Physiol Scand Suppl. 1960;49(169):1–92. Epub 1960/01/01. . 13794892

[31] Lang RM, Badano LP, Mor-Avi V, Afilalo J, Armstrong A, Ernande L, et al. Recommendations for cardiac chamber quantification by echocardiography in adults: an update from the American Society of Echocardiography and the European Association of Cardiovascular Imaging. J Am Soc Echocardiogr. 2015;28(1):1–39 e14. Epub 2015/01/07. doi: 10.1016/j.echo.2014.10.003 . 25559473

[32] Nagueh SF, Smiseth OA, Appleton CP, Byrd BF 3rd, Dokainish H, Edvardsen T, et al. Recommendations for the Evaluation of Left Ventricular Diastolic Function by Echocardiography: An Update from the American Society of Echocardiography and the European Association of Cardiovascular Imaging. Eur Heart J Cardiovasc Imaging. 2016;17(12):1321–60. Epub 2016/07/17. doi: 10.1093/ehjci/jew082 . 27422899

[33] Pellikka PA, Nagueh SF, Elhendy AA, Kuehl CA, Sawada SG, American Society of E. American Society of Echocardiography recommendations for performance, interpretation, and application of stress echocardiography. J Am Soc Echocardiogr. 2007;20(9):1021–41. Epub 2007/09/04. doi: 10.1016/j.echo.2007.07.003 . 17765820

[34] Sicari R, Nihoyannopoulos P, Evangelista A, Kasprzak J, Lancellotti P, Poldermans D, et al. Stress echocardiography expert consensus statement: European Association of Echocardiography (EAE) (a registered branch of the ESC). Eur J Echocardiogr. 2008;9(4):415–37. Epub 2008/06/27. doi: 10.1093/ejechocard/jen175 . 18579481

[35] Houstis NE, Lewis GD. Causes of exercise intolerance in heart failure with preserved ejection fraction: searching for consensus. J Card Fail. 2014;20(10):762–78. Epub 2014/08/12. doi: 10.1016/j.cardfail.2014.07.010 . 25108084

[36] Higginbotham MB, Morris KG, Williams RS, McHale PA, Coleman RE, Cobb FR. Regulation of stroke volume during submaximal and maximal upright exercise in normal man. Circ Res. 1986;58(2):281–91. Epub 1986/02/01. doi: 10.1161/01.res.58.2.281 . 3948345

[37] Del Torto A, Corrieri N, Vignati C, Gentile P, Cattadori G, Paolillo S, et al. Contribution of central and peripheral factors at peak exercise in heart failure patients with progressive severity of exercise limitation. Int J Cardiol. 2017;248:252–6. Epub 2017/09/26. doi: 10.1016/j.ijcard.2017.07.071 . 28942874

[38] Dhakal BP, Malhotra R, Murphy RM, Pappagianopoulos PP, Baggish AL, Weiner RB, et al. Mechanisms of exercise intolerance in heart failure with preserved ejection fraction: the role of abnormal peripheral oxygen extraction. Circ Heart Fail. 2015;8(2):286–94. Epub 2014/10/26. doi: 10.1161/CIRCHEARTFAILURE.114.001825 ; PubMed Central PMCID: PMC5771713. 25344549 PMC5771713

[39] Smarz K, Tysarowski M, Zaborska B, Pilichowska-Paszkiet E, Sikora-Frac M, Budaj A, et al. Chronotropic Incompetence Limits Aerobic Exercise Capacity in Patients Taking Beta-Blockers: Real-Life Observation of Consecutive Patients. Healthcare. 2021;9(2):212. doi: 10.3390/healthcare9020212 33669448 PMC7920432

[40] Lazzeroni D, Castiglioni P, Bini M, Faini A, Camaiora U, Ugolotti PT, et al. Improvement in aerobic capacity during cardiac rehabilitation in coronary artery disease patients: Is there a role for autonomic adaptations? European Journal of Preventive Cardiology. 2017;24(4):357–64. doi: 10.1177/2047487316681341 . 27895211

[41] Conraads VM, Pattyn N, De Maeyer C, Beckers PJ, Coeckelberghs E, Cornelissen VA, et al. Aerobic interval training and continuous training equally improve aerobic exercise capacity in patients with coronary artery disease: the SAINTEX-CAD study. Int J Cardiol. 2015;179:203–10. Epub 2014/12/03. doi: 10.1016/j.ijcard.2014.10.155 . 25464446

[42] Takagi S, Murase N, Kime R, Niwayama M, Osada T, Katsumura T. Aerobic training enhances muscle deoxygenation in early post-myocardial infarction. Eur J Appl Physiol. 2016;116(4):673–85. Epub 2016/01/14. doi: 10.1007/s00421-016-3326-x ; PubMed Central PMCID: PMC4819748. 26759155 PMC4819748

[43] Podolec P, Rubis P, Tomkiewicz-Pajak L, Kopec G, Tracz W. Usefulness of the evaluation of left ventricular diastolic function changes during stress echocardiography in predicting exercise capacity in patients with ischemic heart failure. J Am Soc Echocardiogr. 2008;21(7):834–40. Epub 2008/01/29. doi: 10.1016/j.echo.2007.12.008 . 18222643

[44] Fontes-Carvalho R, Sampaio F, Teixeira M, Rocha-Gonçalves F, Gama V, Azevedo A, et al. Left ventricular diastolic dysfunction and E/E’ ratio as the strongest echocardiographic predictors of reduced exercise capacity after acute myocardial infarction. Clin Cardiol. 2015;38(4):222–9. Epub 2015/02/25. doi: 10.1002/clc.22378 ; PubMed Central PMCID: PMC6711017. 25707582 PMC6711017

[45] Guazzi M, Generati G, Borlaug B, Alfonzetti E, Sugimoto T, Castelvecchio S, et al. Redistribution of cardiac output during exercise by functional mitral regurgitation in heart failure: compensatory O(2) peripheral uptake to delivery failure. Am J Physiol Heart Circ Physiol. 2020;319(1):H100–h8. Epub 2020/05/23. doi: 10.1152/ajpheart.00125.2020 . 32442022

[46] Sugimoto T, Bandera F, Generati G, Alfonzetti E, Barletta M, Losito M, et al. Left Atrial Dynamics During Exercise in Mitral Regurgitation of Primary and Secondary Origin: Pathophysiological Insights by Exercise Echocardiography Combined With Gas Exchange Analysis. JACC Cardiovasc Imaging. 2020;13(1 Pt 1):25–40. Epub 2019/03/18. doi: 10.1016/j.jcmg.2018.12.031 . 30878440

[47] Nedeljkovic I, Banovic M, Stepanovic J, Giga V, Djordjevic-Dikic A, Trifunovic D, et al. The combined exercise stress echocardiography and cardiopulmonary exercise test for identification of masked heart failure with preserved ejection fraction in patients with hypertension. Eur J Prev Cardiol. 2016;23(1):71–7. Epub 2015/09/12. doi: 10.1177/2047487315604836 . 26358991

[48] Bandera F, Generati G, Pellegrino M, Garatti A, Labate V, Alfonzetti E, et al. Mitral regurgitation in heart failure: insights from CPET combined with exercise echocardiography. Eur Heart J Cardiovasc Imaging. 2017;18(3):296–303. Epub 2016/05/20. doi: 10.1093/ehjci/jew096 . 27194781

[49] Smarz K, Zaborska B, Jaxa-Chamiec T, Budaj A. Exercise left ventricular outflow tract obstruction as a cause of exercise intolerance: combined stress echocardiography and cardiopulmonary exercise testing. Kardiol Pol. 2018;76(10):1492. Epub 2018/10/20. doi: 10.5603/KP.2018.0209 . 30338833

[50] Thaden JJ, McCully RB, Kopecky SL, Allison TG. Echocardiographic determinants of peak aerobic capacity and breathing efficiency in patients with undifferentiated dyspnea. Am J Cardiol. 2014;114(3):473–8. Epub 2014/06/21. doi: 10.1016/j.amjcard.2014.04.054 . 24948490

[51] Kitzman DW, Higginbotham MB, Cobb FR, Sheikh KH, Sullivan MJ. Exercise intolerance in patients with heart failure and preserved left ventricular systolic function: failure of the Frank-Starling mechanism. J Am Coll Cardiol. 1991;17(5):1065–72. Epub 1991/04/01. doi: 10.1016/0735-1097(91)90832-t . 2007704

[52] Yancy CW, Jessup M, Bozkurt B, Butler J, Casey DE Jr., Drazner MH, et al. 2013 ACCF/AHA guideline for the management of heart failure: a report of the American College of Cardiology Foundation/American Heart Association Task Force on Practice Guidelines. J Am Coll Cardiol. 2013;62(16):e147–239. Epub 2013/06/12. doi: 10.1016/j.jacc.2013.05.019 . 23747642

