# Correction: Combined use of stress echocardiography and cardiopulmonary exercise testing to assess exercise intolerance in patients treated for acute myocardial infarction

**DOI:** 10.1371/journal.pone.0307152

**Published:** 2024-07-10

**Authors:** Krzysztof Smarz, Tomasz Jaxa-Chamiec, Beata Zaborska, Maciej Tysarowski, Andrzej Budaj

The Data Availability statement for this paper is incorrect. The correct Data Availability statement is: “The complete raw dataset for this study is publicly available from the Mendeley Data repository (http://doi.org/10.17632/dphcf83ty6).”).

In Table 1, the data in "Hemoglobin at discharge, g/dL" are also incorrectly formatted. This data should have appeared in one row. Please see the correct Table 1 here.

**Table 1:** Demographic and clinical characteristics during hospitalization for acute myocardial infarction according to percentage of predicted oxygen uptake (Group 1, < 50%; Group 2, 50–74%; Group 3, 75–99%; and Group 4, ≥100%).

Variables	Total	Group 1	Group 2	Group 3	Group 4	p*
	(n = 81)	(n = 7)	(n = 41)	(n = 24)	(n = 9)	
**Demographics**						
Male sex, n (%)	57 (70)	6 (86)	31 (76)	18 (75)	2 (22)	0.045^a^
Age, years	57.6 ± 11.0	58.8 ± 13.6	56.8 ± 9.4	57.1 ± 12.9	61.8 ± 10.9	0.606
Body mass index, kg/m^2^	28 ± 4	29 ± 5	28 ± 4	27 ± 4	24 ± 3	0.011^b^
**Comorbidity, n (%)**						
Current smoking	38 (47)	5 (71)	25 (61)	6 (25)	2 (22)	0.066
Hypertension	54 (67)	5 (71)	27 (66)	15 (62)	7 (78)	1
Hyperlipidemia	63 (78)	6 (86)	34 (83)	16 (67)	7 (78)	1
Diabetes mellitus/Impaired glucose tolerance	25 (31)	0 (0)	13 (32)	8 (33)	4 (44)	0.874
Paroxysmal atrial fibrillation	2 (2)	0 (0)	1 (2)	1 (4)	0 (0)	1
Physical activity before myocardial infarction						
Low	17 (21)	4 (57)	6 (15)	6 (25)	1 (11)	0.239
Moderate	41 (51)	3 (43)	24 (59)	10 (42)	4 (44)	1
High	23 (28)	0 (0)	11 (27)	8 (33)	4 (44)	0.874
**Clinical characteristics**						
STEMI, n (%)	38 (47)	3 (43)	21 (51)	11 (46)	3 (33)	1
Non STEMI, n (%)	43 (53)	4 (57)	20 (49)	13 (54)	6 (67)	1
Culprit lesion, n (%)						
Right coronary artery	20 (25)	1 (14)	13 (32)	4 (17)	2 (22)	1
Left anterior descending artery	47 (58)	2 (28)	22 (54)	12 (50)	7 (78)	1
Circumflex artery	19 (23)	5 (71)	6 (15)	6 (25)	2 (22)	1
Troponin T the highest value, ng/L, median (IQR)	784 (242, 2216)	2380 (571, 4107)	784 (204, 3225)	859 (450, 1777)	242 (162, 828)	1
Hemoglobin at discharge, g/dL	14.05 ± 1.21	14.19 ± 1.28	14.21 ±1.21	13.95 ± 1.14	13.50 ± 1.40	0.386
Creatinine clearance at discharge, mL/min	108 ± 30	113 ± 52	113 ± 28	104 ± 24	92 ± 29	0.245
LVEF at discharge, %, median (IQR)	55 (50, 60)	60 (55, 60)	52 (46, 57)	55 (50, 58)	60 (60, 60)	0.380
**Medication at discharge,** n (%)						
ACE-I/ARB	78 (96)	7 (100)	39 (95)	23 (96)	9 (100)	1
Beta-blocker	70 (86)	6 (86)	36 (88)	20 (83)	8 (89)	1
Aspirin	80 (99)	7 (100)	40 (98)	24 (100)	9 (100)	1
P2Y_12_ inhibitors	79 (97)	7 (100)	39 (95)	24 (100)	9 (100)	1
Statin	79 (98)	7 (100)	40 (98)	23 (96)	9 (100)	1
Nitrate	4 (5)	0 (0)	1 (2)	2 (8)	1 (11)	1
Calcium channel blocker	19 (23)	4 (57)	8 (20)	5 (21)	2 (22)	0.590
Diuretic	22 (27)	2 (29)	12 (29)	5 (21)	3 (33)	1

*Note*: *Values represent mean* ± *SD*, *median (IQR) or number (%)*. *P2Y*_*12*_ *inhibitors*: *clopidogrel or ticagrelor*. *Creatinine clearance was calculated according to Cockroft-Gault equation*.

Abbreviations: ACE-I, angiotensin converting enzyme inhibitors; ARB, angiotensin receptor blockers; CPET-SE, combined stress echocardiography and cardiopulmonary exercise testing; LVEF, left ventricular ejection fraction; STEMI, acute myocardial infarction with ST segment elevation.

* presented non-significant p value is the lowest from Bonferroni post hoc test (by Group).

Bonferroni post hoc test (by Group) for significant p value:

^*a*^*Group 2 vs*. *Group 4;*

^*b*^*Group 2 vs*. *Group 3;*

* for non-significant presented the lowest p value
