# Supplementary material for: Combined use of stress echocardiography and cardiopulmonary exercise testing to assess exercise intolerance in patients treated for acute myocardial infarction
Source: PLoS One. 2021 Aug 5;16(8):e0255682. doi: 10.1371/journal.pone.0255682 (PMC8341484; doi:10.1371/journal.pone.0255682)
Supplement: S1 Table — Abbreviations: A, late mitral inflow velocity; A-VO2Diff, arteriovenous oxygen difference; DBP, diastolic blood pressure; E, early mitral inflow velocity; e’, early diastolic myocardial velocity; LV EF, left ventricular ejection fraction; LV EDV, left ventricular end-diastolic volume; LV ESV, left ventricular end-systolic volume; LV s’, left ventricular systolic myocardial velocity; ΔO2/ΔWR, oxygen uptake to work rate increment; RV s’, right ventricular systolic myocardial velocity; SBP, systolic blood pressure; TAPSE, tricuspid annulus plane systolic excursion; VE/VCO2 slope, minute ventilation to carbon dioxide production slope; VO2, oxygen uptake; WMSI, wall motion score index. (DOCX) [file pone.0255682.s001.docx]

**S1 Table. Spearman correlations for peak oxygen uptake, percent predicted oxygen uptake and clinical, and combined stress echocardiography and cardiopulmonary exercise testing data.**

| **Variable** | **Spearman correlations with peak VO_2_, mL/kg/min** | **Spearman correlations with percent predicted VO_2_, %** |
| --- | --- | --- |
| Age, years | -0.26 | 0.14 |
| Body mass index, kg/m2 | -0.22 | -0.23 |
| Troponin T the highest value, ng/L | -0.06 | -0.12 |
| Hemoglobin at discharge, g/dL | 0.13 | -0.11 |
| Creatinine clearance at discharge, mL/min | 0.19 | -0.18 |
| SBP at peak, mmHg | 0.07 | 0.10 |
| DBP at peak, mmHg | -0.20 | -0.20 |
| Heart rate at rest, bpm | -0.04 | 0.01 |
| Heart rate at peak, bpm | 0.43 | 0.37 |
| Percent predicted heart rate, % | 0.28 | 0.46 |
| Chronotropic index, % | 0.37 | 0.44 |
| ∆O_2_/∆WR, mL/min/watt | 0.38 | 0.53 |
| VE/VCO_2_ slope | -0.47 | -0.25 |
| Stroke volume at rest, mL | -0.03 | -0.09 |
| Stroke volume at peak, mL | 0.12 | 0.03 |
| WMSI at rest | -0.10 | -0.21 |
| WMSI at peak | -0.13 | -0.23 |
| LV EF at rest, % | -0.18 | -0.12 |
| LV EF at peak, % | 0.03 | 0.05 |
| LV EDV at rest, mL | 0.22 | -0.07 |
| LV EDV at peak, mL | 0.09 | -0.09 |
| LV ESV at rest, mL | 0.23 | 0.01 |
| LV ESV at peak, mL | 0.01 | -0.10 |
| LV s' at rest, cm/s | 0.20 | -0.12 |
| LV s' at peak, cm/s | 0.36 | 0.07 |
| TAPSE at rest, cm | 0.02 | -0.04 |
| TAPSE at peak, cm | 0.16 | 0.02 |
| RV s' at rest, cm/s | -0.01 | 0.08 |
| RV s' at peak, cm/s | 0.21 | 0.19 |
| E/A ratio at rest | 0.13 | -0.08 |
| E/A ratio at peak | 0.16 | 0.11 |
| e' at rest, cm/s | 0.23 | -0.05 |
| e' at peak, cm/s | 0.25 | 0.06 |
| E/e' ratio at rest | -0.20 | 0.11 |
| E/e' ratio at peak | -0.02 | 0.22 |
| A-VO2Diﬀ at rest, mL/dL | 0.38 | 0.15 |
| A-VO2Diﬀ at peak, mL/dL | 0.68 | 0.36 |

Abbreviations: A, late mitral inflow velocity; A-VO2Diﬀ, arteriovenous oxygen difference; DBP, diastolic blood pressure; E, early mitral inflow velocity; e', early diastolic myocardial velocity; LV EF, left ventricular ejection fraction; LV EDV, left ventricular end-diastolic volume; LV ESV, left ventricular end-systolic volume; LV s', left ventricular systolic myocardial velocity; ∆O_2_/∆WR, oxygen uptake to work rate increment; RV s', right ventricular systolic myocardial velocity; SBP, systolic blood pressure; TAPSE, tricuspid annulus plane systolic excursion; VE/VCO_2_ slope, minute ventilation to carbon dioxide production slope; VO_2_, oxygen uptake; WMSI, wall motion score index.
