# Supplementary material for: Combined use of stress echocardiography and cardiopulmonary exercise testing to assess exercise intolerance in patients treated for acute myocardial infarction
Source: PLoS One. 2021 Aug 5;16(8):e0255682. doi: 10.1371/journal.pone.0255682 (PMC8341484; doi:10.1371/journal.pone.0255682)
Supplement: S2 Table — Patient A is an obese man with low physical activity, bad peripheral oxygen extraction, and low exercise capacity; patient B is a normal weight woman with high levels of daily physical activity, good peripheral oxygen extraction, and good exercise capacity. Abbreviations: A-VO2Diff, arteriovenous oxygen difference; BMI, body mass index; DBP, diastolic blood pressure; LV EF, left ventricular ejection fraction; SBP, systolic blood pressure; VE/VCO2 slope, minute ventilation to carbon dioxide production slope; VO2, oxygen uptake. (DOCX) [file pone.0255682.s002.docx]

**S2 Table. Summary of the combined stress echocardiography and cardiopulmonary exercise testing parameters proposed to functional phenotyping on the base of two patients from the study.**

|  | **Patient A** | **Patient B** |
| --- | --- | --- |
| Age, years | 61 | 65 |
| BMI kg/m^2^ | 32 | 25 |
| LV EF at rest, % | 70 | 59 |
| Peak VO_2_, mL/kg/min | 13.6 | 27.0 |
| Percent predicted VO_2_, % | 49 | 144 |
| RER | 1.05 | 1.05 |
| VE/VCO_2_ slope | 25 | 19 |
| Heart rate at peak, bpm | 106 | 105 |
| Percent predicted heart rate, % | 67 | 68 |
| SBP at peak, mmHg | 220 | 190 |
| DBP at peak, mmHg | 80 | 60 |
| Stroke volume at peak, mL | 124 | 78 |
| Stroke volume index at peak, mL/m^2^ | 47 | 44 |
| A-VO_2_Diﬀ at peak, mL/dL | 11.3 | 22.9 |

Patient A is an obese man with low physical activity, bad peripheral oxygen extraction, and low exercise capacity, patient B it is a woman in normal weight with high every day physical activity, good peripheral oxygen extraction, and good exercise capacity.

Abbreviations: A-VO_2_Diﬀ, arteriovenous oxygen difference; BMI, body mass index; DBP, diastolic blood pressure; LV EF, left ventricular ejection fraction; SBP, systolic blood pressure; VE/VCO_2_ slope, minute ventilation to carbon dioxide production slope; VO_2_, oxygen uptake.
